# Polycotyly: How Little Do We Know?

**DOI:** 10.3390/plants13081054

**Published:** 2024-04-09

**Authors:** Yong-Bi Fu

**Affiliations:** Plant Gene Resources of Canada, Saskatoon Research and Development Centre, Agriculture and Agri-Food Canada, 107 Science Place, Saskatoon, SK S7N 0X2, Canada; yong-bi.fu@agr.gc.ca

**Keywords:** cotyledon number, tricotyledon, polycotyledon, cotyledon development, polycotyly origin, polycotyly genetics, polycotyly evolution, polycotyly breeding

## Abstract

Polycotyly, an interesting characteristic of seed-bearing dicotyledonous plants with more than two cotyledons, represents one of the least explored plant characters for utilization, even though cotyledon number was used to classify flowering plants in 1682. Gymnosperm and angiosperm species are generally known to have one or two cotyledons, but scattered reports exist on irregular cotyledon numbers in many plant species, and little is known about the extent of polycotyly in plant taxa. Here, we attempt to update the documentation of reports on polycotyly in plant species and highlight some lines of research for a better understanding of polycotyly. This effort revealed 342 angiosperm species of 237 genera in 80 (out of 416) families and 160 gymnosperm species of 26 genera in 6 (out of 12) families with reported or cited polycotyly. The most advanced research included the molecular-based inference of the phylogeny of flowering plants, showing a significant departure from the cotyledon-based classification of angiosperm plants, and the application of genetic cotyledon mutants as tools to clone and characterize the genes regulating cotyledon development. However, there were no reports on breeding lines with a 100% frequency of polycotyly. Research is needed to discover plant species with polycotyly and to explore the nature, development, genetics, evolution, and potential use of polycotyly.

## 1. Introduction

Polycotyly is an interesting characteristic of seed-bearing dicotyledonous plants with more than two cotyledons, but it may represent one of the least explored plant characteristics for utilization. Cotyledons have been long known to play an important role in plant classification [1]. Biologically, a cotyledon is a significant part of the embryo within a plant seed, serving in some species as the stored food reserve for seed germination and the first photosynthetic organ of a plant [2]. Over the last two centuries, researchers have observed plant seedlings in dicotyledonous plants that have more than the expected two cotyledons, collectively referred to as pleiocotyly (or extra cotyledons) [3]. The occurrence of extra cotyledons was commonly treated as a rare and trivial abnormality [4]. Several teratological pleiocotylous features were described, and many terms were created: syncot (a fused cotyledon), schizocot (a split cotyledon), (pseudo- or syn-) monocot (a single or fused cotyledonary tube or cup), tricot (three cotyledons), tetracot (four cotyledons), and polycot (many cotyledons) [5,6]. In this paper, we focus more on the variation in cotyledon numbers and less on the teratological aspect of cotyledons, and the term polycotyly will be used throughout.

Gymnosperm and angiosperm species are generally known to have one or two cotyledons. However, scattered reports exist on irregular cotyledon numbers in plant species over the last two centuries, and there is no comprehensive account of the extent of polycotyly in seed-bearing plant species. The first documentation of polycotyly for many plant species was made by Duchartre [7], followed by other researchers such as von Mueller [8] and Guppy [9]. Several summaries of plant species with polycotyly in the 20th century are also found [10,11,12,13,14]. Specifically, Penzig [15] recorded polycotyly for approximately 145 species, and Reynard [16] claimed there were 295 species of 68 families with reported polycotyly, but the claimed species were not documented. Thus, little is known about the scope and scale of polycotyly and its taxonomic distribution.

There also exist diverse lines of research on polycotyly, including taxonomy, morphology, anatomy, cotyledon development, genetics, and breeding (e.g., [2,3,17]). The observed variations in cotyledons challenged the angiosperm classification (e.g., [7]) and posed questions on the origin of monocots (e.g., [17]). The various anatomic features of polycotyly boosted the search for answers on the origin and development of cotyledons (e.g., [18]). Rare occurrences of polycotyly in a species provided a base for de Vries [19,20] to configure and evaluate his mutation theory. The revealed polycotylous phenotypes raised questions on inheritance [4]. However, how much is known about polycotyly as a quantitative character (first suggested by Bexon [21]) remains an unanswered question of interest. The possible agronomic benefits of using polycotyly have not been adequately explored [22].

In this review, we attempt to update the documentation of reports on polycotyly in plant species, highlight some lines of polycotyly research over the last two centuries, and present our thoughts on further research that can advance our knowledge about polycotyly. We hope that this review can allow for a better understanding of the extent of polycotyly occurrences in the taxonomic distribution of seed-bearing plant species and of polycotyly as a quantitative character [21].

## 2. The Extent of Polycotyly in Gymnosperms and Angiosperms

To update the documentation of polycotyly occurrences, we undertook a literature search on reports of plant species with polycotyly, largely based on the citations of early or recent related reports or using different words or phrases for Google searches. Several papers and books were found to report and/or summarize polycotyly occurrences. Duchartre [7] seems to have been the first to document plant species with polycotyly, followed by other researchers like von Mueller [8] reporting polycotyly in *Persoonia* and Guppy [9] reporting the occurrence of polycotyly in 12 angiosperm species in 10 families. Krause [23], Gain [24], de Vries [10], and Guillaumin [25] also summarized the records of polycotyly. For angiosperms, the most comprehensive records were found in the descriptions of teratological phenomena by Penzig [15], with approximately 145 species, and in the summary by Reynard [16] of 295 species of 68 families (without listing the claimed species). For gymnosperms, there were several major papers summarizing the occurrences of polycotyly [11,12,13,14]. Scattered reports on the subject from the 1930s to 2023 were also found.

The search helped to update the documentation on 502 species representing 263 genera of 86 (out of 428) families with reported polycotyly (Supplementary Materials Section A). Specifically, there were 342 angiosperm species of 237 genera in 80 (out of 416) families and 160 gymnosperm species of 26 genera in 6 (out of 12) families with polycotyly, as shown in Table 1. A note for Table 1 is that the angiosperm family name is updated following the angiosperm phylogeny group classification IV [26] and the number of known species for a family was acquired from Christenhusz and Byng [27]. The angiosperm families Fabaceae, Asteraceae, and Proteaceae have recorded 39, 27, and 25 species, respectively, while the gymnosperm Pinaceae and Cupressaceae families have 122 and 31 species, respectively. Thus, polycotyly occurs in a wide range of taxonomic families, while the proportions of species with the recorded polycotyly are considerably low when considering that the extant relevant gymnosperms and angiosperms contain 618 and 171,983 known species, respectively (Table 1). Note that the update was based on 154 references with reported and cited polycotyly from many scientific journals or publications from 1848 to 2023, and many of those were the first report for the species (Supplementary Materials Section B).

Several features of polycotyly occurrences were observed from the acquired reports. First, the number of cotyledons in the reported gymnosperms ranged widely from 3 to 24, while the reported angiosperms largely had 3 to 4 cotyledons (Supplementary Materials Section A). Second, for those reports with spontaneous occurrences, the observed frequencies were generally up to 6% of the evaluated seedlings in a species [9,28], although the occurrence frequencies were not recorded in this documentation due to the lack of environmental records. Third, polycotyly can occur spontaneously in natural populations (e.g., [22]), in polluted sites (e.g., [29]), or in common gardens (e.g., [19,20]) and can be induced by hybridization (e.g., [30]), by mutagenesis (e.g., [31,32]), or on tissue culture media (e.g., [33]). Differences in cotyledon count were also reported in different environments [34,35] and seasons [18] and in the offspring of the fruits collected from different tomato branch positions [36].

The most distinguishing feature of polycotyly occurrences was presented in the gymnosperm Pinaceae with 122 (out of 250) known species and the angiosperm Proteaceae with 25 (out of 1660) species (Table 1). Pinaceae has polycotyly occurrences in 8 of the 11 genera, with *Pinus* having 3 to 24 cotyledons in 62 species and *Abies* having 3 to 13 cotyledons in 23 species (Supplementary Materials Section A). *Pinus maximartinezii* Rzedowski has 18 to 24 cotyledons (Supplementary Materials Section A). Similarly, Proteaceae has polycotyly in 2 of the 83 genera, with *Persoonia* having 3 to 8 cotyledons in 24 species and *Isopogon* having 3 cotyledons in 1 species (Supplementary Material Section A). *Persoonia quinquenervis* Hook. and *Persoonia teretifolia* R.Br. have 7 to 8 cotyledons. Why these two genera *Pinus* and *Persoonia* are so unique with so many cotyledons in seed-bearing plants remains a research question of great interest in plant biology.

It is worth noting that the updated documentation may represent only the low bound for the reported species with polycotyly, as reports published in old journals or different languages were not necessarily accessible nor verifiable. Also, our search efforts cannot be considered to be exhaustive, and thus some reports may have not been included. Some reported species had synonyms and classification changes, complicating the documentation. When there were multiple reports on the same species, we recorded the earliest reports possible. For species with multiple reports of various sources (spontaneously or from hybridization, mutagenesis, somatic culture), we reported spontaneous polycotyly first, followed by the first report of other kinds. The update considered only the cotyledon count for each report, as the acquired reports over the two centuries varied in the other polycotyly features. Moreover, some reports based on polycotylous embryos may have misinterpreted the endosperm as part of an embryo, as illustrated for *Psittacanthus* spp. by Gonzalez and Pabon-Mora [37].

## 3. Advances in Polycotyly Research

Cotyledons are known to play an important role in plant classification. John Ray [1] was the first to adopt the idea of using seed and embryo characters [38] to classify plant species and to separate flowering plants into monocots (or Monocotyledons) and dicots (or Dicotyledons) based on cotyledon number. However, arguments against such a taxonomic division were not lacking (e.g., see the review of Bancroft [17]), as the cotyledon number was later found to vary greatly in flowering plants [7]. Some dicots have no embryonic leaves, one cotyledon, or even three or four cotyledons, while some monocots have more than one cotyledon (e.g., [39]). Plants in the gymnosperm group generally have two cotyledons, but many cotyledons in a species are also observed. It was such a cotyledon variation that has been the subject of research over the past two centuries. In what follows, we will highlight five lines of research on polycotyly.

### 3.1. Phylogenetics

Cotyledon-based classification of flowering plants [1] is simple and natural when compared with later classification systems [17] that were established by Linnaeus [40] and others (e.g., [41]) based on complex morphological characters (e.g., those associated with pollen and xylem). However, such a simple distinction of seed-bearing plants is limited to describing ancestral affinity or the evolutionary origin of angiosperm groups. This limitation is largely reflected in the central argument on the evolutionary position of monocots in seed-bearing plants [17]. The early findings of large variations present in cotyledon numbers and the morphologies within and between monocots and dicots were unable to elucidate consistently the origin of monocots and dicots, separating many botanists. It was Sargent [18] who generated some empirical support to advance the idea of Strasburger [42] that monocots are derived from dicotyledonous stock. Based on the anatomical investigation of the fusion of two cotyledons to form one member (or syncotyly), Sargent [18] was convinced that the symmetric pattern of plant vascular tissues in monocotyledonous seedlings may be referred to as the same as that of dicotyledonous seedlings. A literature review by Haines and Lye [43] showed evidence for supporting the syncotylar theory of monocots’ origin, which is consistent with the early assessment of Takhtajan [44]. However, arguments against this theory also existed [45,46,47]. For example, based on various criteria for homology, Burger [47] concluded from his literature review that “the cotyledons of monocots and dicots are fundamentally different structures that do not share a common phylogenetic origin”.

Angiosperms are more complex in character than fits the original cotyledon-based classification. Angiosperm species are diverse in their genome size and organization, reproductive morphology, and chemistry, among other things, but they share a suite of synapomorphies [48]. Phylogenetic studies using molecular tools in the 1990s and 2000s (e.g., [49]) largely discredited the old cotyledon-based classification of ancestral affinity and revealed the phylogeny of angiosperm plants, showing a significant departure from cotyledon-based classification. Specifically, molecular-based inferences of angiosperm phylogeny have established a mono-phylogenetic group called monocots (which comprises all the monocot species from the old classification), another group called eudicots (which include most of the species in the original dicots), and other clades that comprise a large diversity of flowering plants [48]. Our current understanding still holds that monocots may be derived from some primitive eudicots [50], as molecular evidence has shown that monophyletic monocots are related to magnoliids, eudicots, Chloranthaceae, and Ceratophyllaceae [51]. However, the exact position of monocots in the evolution of angiosperms remains a topic of research (e.g., [52]).

Gymnosperms generally have two cotyledons [53]. Our update on the cotyledon count revealed 6 (out of 12) families with polycotylous species, including the family of Sciadopityaceae (Table 1). The family Pinaceae would have average cotyledon numbers ranging from a low of three in *Tsuga* to a high of nine in *Cedrus* (Supplementary Materials Section A). The number of cotyledons in the reported gymnosperms ranges widely from 3 to 24. Such wide variation is consistent with our general knowledge that the cotyledon number is not a characteristic species-specific number, particularly in conifers. Thus, it is not surprising that the recently updated gymnosperm classification (e.g., [54]) did not consider the number of cotyledons as a valuable character but focused on the phylogeny inferred from molecular data [55].

### 3.2. Cotyledon Morphology, Anatomy, and Development

Many studies on the morphology and anatomy of abnormal seedlings were conducted, particularly before 1940, to understand the ancestral affinity of monocots and dicots, as described in the above section, and the origin and development of cotyledons [56,57]. A large diversity in cotyledon morphology across seed plants was found (e.g., [5,6,56]). Such diversity was rarely associated with cotyledon number but largely with variations in their development. The occurrence of various degrees of aberrant or teratological cotyledons, such as syncotyly, pseudomoncotyly, schizocotyly, and polycotyly, has been well documented in the literature. An extreme case was the observation of heterocotyly, syncotyly, amphisyncotyly, lateral syncotyly, pseudomonotyly, malformation, schizocotyly, hemitricotyly, tricotyly, hemitetracotyly, and tetracotyly in about 2000 seedlings of *Acer pseudoplatanus* L. [6]. Cotyledons also display variations in size measured as isocotyly, anisocotyly, microcotyledons, and macrocotyledons (e.g., [58]). Such diverse cotyledon morphologies were considered to be abnormal and provided few answers, but revealed complexities, in terms of our understanding of the modes of increases and decreases in cotyledon number [5]. It also became obvious that classical morphology analysis has its limits, as continuum and process morphologies need to be considered to analyze cotyledon complexity [59]. The idea of evolutionary developmental morphology (e.g., [60]) may be applied to analyzing cotyledon morphological variation. 

A large number of anatomic studies on teratological seedlings were undertaken to elucidate obscure morphological problems for a better understanding of cotyledons’ origins (e.g., [5,11,12,13,18,21,61,62,63,64,65,66]). It was found that the modes of increasing or decreasing the number of cotyledons in a species could be explained by cotyledon fusion, loss, or fission [5,18,62]. For example, cotyledon fusion was proposed to explain the observed syncotyly (or single cotyledons in angiosperms) as the origin of monocots from a primitive dicot [5,18]. Hill and de Fraine [11,12,13] explained the observed schizocotyly (or polycotyly in conifers) as the majority of polycotyledonous conditions being derived from dicotyledonous conditions through the splitting of seed leaves. In other words, these researchers assumed that dicotyledony was a primitive condition in flowering plants and explained the observed variation in cotyledon count as the single cotyledon of a dicotyledonous seedling being derived from two fused cotyledons, while polycotyledony was the result of a deep division of cotyledons. These explanations provided some support not only for understanding the evolutionary origin of monocots, as described in the above section, but also cotyledons’ origins. However, there were arguments for (e.g., [6]) and against these explanations (e.g., [46,47]). Two observations incompatible with these explanations are that the cotyledons of monocots and dicots also differ in form, with no true intermediates, and that the third leaf of Nymphaealean seedlings seems to be identical to the single cotyledon of monocots [46,47]. More interestingly, the revealed modes of cotyledon development were found to be associated with variations among fruits (e.g., [36]), plants (e.g., [67]), environments (e.g., [34,35]), and geographic habits (e.g., [18]). These associations imply that the reported cotyledon variations may have been generated through different developmental mechanisms, which remains poorly understood [68].

Better insights into the development of cotyledons as the first aerial organs were gained with the explosion in the use of induced genetic mutants with defective cotyledon development as tools for gene cloning in the 1990s [69,70,71,72,73]. Briefly, genetic mutants were generated via ethyl methane sulfonate mutagenesis of seeds and isolated through the screening of thousands of mutagenized seedlings with specific defective cotyledon phenotypes, followed by genetic crosses and testing with their wild types (e.g., [71]). Genes were cloned and characterized with various molecular tools such as recombination mapping (e.g., [72,74]). Many genetic mutants with defective cotyledons were identified and characterized in *Arabidopsis* and other species, and relevant genes controlling some of these mutants were also cloned, as reviewed by Chandler [2]. Many genetic pathways in cotyledon development were elucidated. These studies confirmed that there exists a small number of orthologous gene hierarchies regulating cotyledon development, particularly those involving auxin. Learning from *Arabidopsis* mutants, there are two major features of the identified genes. The first is redundancy among several gene families such as *Pin* [74] and transcription factors such as *DRN*/*DRNL* [75]. Such redundancy reflects the genetic robustness and evolutionary adaptation to ensure buffering against mutations. The other is the conservation of regulatory pathways such as the *STM* pathway involving auxin. However, we are still far from understanding the patterns and processes of cotyledon development (e.g., [2,76,77]), as little is known about the conservation and divergence of these regulatory pathways in other species.

### 3.3. Genetics

The earliest inheritance study of polycotyly as a quantitative character can be traced back to those selections made by de Vries [19,20] of *Helianthus annuus* syncots, in which the proportion of syncots gradually increased over four generations, implying the studied syncot was a heritable trait. Increases in polycotylous seedlings over generations of selections were also observed in *Brassica* [4] and other species [78,79]. Together, these studies confirmed that polycotyly is heritable. After re-evaluating the early works of de Vries, Straub [78] reasoned that tricotyly was controlled by one recessive gene, with its expression modified by two dominant suppressors and the environment. Specifically for the latter, increases in tricot seedlings through selection were found to be higher when seeding in the fall than in the spring and early summer. Similar studies in tomato and rapeseed (*Brassica napus* L.) also showed many other factors contributed to the expression of tricotyly. For example, Haskell [36] reported that the percentage of tomato tricots produced by fruits at the various truss positions (or the positions of a flower cluster) decreased from the first to the third truss position and increased from the fourth. The tomato tricot percentages were found to vary from plant to plant and fruit to fruit [67]. More split cotyledons of rapeseed were found in the seeds of plants grown at 16–21 °C than those grown at 21–26 °C [35]. Thus, both genetics and the environment play an important role in the expression of polycotyly. However, little is known about the extent of the contribution epigenetics made to the generation of the observed polycotyly. 

Research also exists on specific genetic analyses of tricotyly in some species. Palmer [35] conducted a genetic analysis of split cotyledons in rapeseed through controlled selfing and crossing and found that there were several genes involved in the expression of split cotyledons. Another interesting finding acquired from the similarity of reciprocal crosses was that the phenotype of an embryo was determined by its genotype and not by the reciprocal parental genotypes. Bray [80] performed a quantitative genetic analysis of polycotyly in alfalfa and found that selection for the abnormal parental phenotype increased the proportion of abnormal progeny in the population studied but did not reduce the additive population variance (compared to normal dicotyledonous seedlings). Guess [81] reported a genetic study of tricotyly in *Haplopappus gracilis* and concluded that the tricotyly observed in this species was controlled by a recessive gene located in the B chromosome and was highly influenced by higher temperatures. Recently, a genetic segregation analysis of tricot and tetracot seedlings in selfed S1 and S2 generations of yellow mustard (*Sinapis alba* L.) suggested that the development of tricot and tetracot seedlings was controlled by a combination of genes at multiple loci [82]. More interestingly, two S2 tetracot plants produced 6.7% and 16.7% tricot, but no tetracot, seedlings [82]. 

With advances in gene cloning via the development of induced genetic mutants with defective cotyledons, many studies have been conducted to identify the genes conditioning the development of cotyledons, particularly in *Arabidopsis* species, over the last three decades (e.g., [70,75,83,84,85]). These research efforts revealed many genes or loci associated with the development of various cotyledon phenotypes in several species, as summarized in Chandler [2]. For example, genes such as *xtc* and *amp1* that can generate polycotyly in *Arabidopsis* were identified [66,80]. These findings further confirmed that the development of polycotyly is genetically complicated and controlled by many genes across different chromosomes. However, it was also reported that *Arabidopsis* tricots can be caused by abnormal chromosome segregation at meiosis [86] and develop in a stochastic manner (e.g., [87]).

From the inheritance studies above, one could conclude that polycotyly is a genetically complex quantitative character, controlled by many genes at multiple loci, and its development can be highly influenced by the environment. There were estimates of heritability for polycotyly at 0.32 in *Cedrus deodara* [88] and 0.53 in Douglas fir [89], but there have been no estimates of heritability for polycotyly in natural populations of angiosperm plants across different environments [90]. Thus, the genetic basis of polycotyly remains poorly understood due to a lack of thorough genetic analyses. We do not know how many genes are involved in the development of polycotyly in plant species with frequent occurrences of polycotyly, where they are located on chromosomes, and what effects they have on development. Answers to these questions may have to be obtained from genomic analyses of polycotyly.

### 3.4. Evolution

A cotyledon is a significant part of the embryo within a plant seed and plays an important role in seedling establishment, seedling survival, and plant growth potential [2]. Soybean cotyledons were shown to increase carbonic anhydrase activity and photosynthesis capability [91], and the artificial removal of peanut cotyledons reduced the plant growth and peanut yield by 15–30% [92]. Cotyledons also contributed to plant growth and hybrid vigor in *Arabidopsis* [93]. Some of the literature also suggests that a cotyledon functions as a haustorial organ with intercalary growth to absorb nutrients from the gymnosperm megagametophyte or angiosperm endosperm (e.g., [94]). This extra cotyledon function is useful for exploring and explaining the origin and evolution of cotyledons per se (e.g., [95]), which represents an interesting topic of botanical research. 

Here, we would pose a simple biological question paid little attention in the past: does polycotyly enhance seedling fitness when compared to plants with one or two cotyledons? Some of the literature seems to suggest an answer YES for conifers and NO for angiosperms. For example, it was found that the usually high photosynthesis rates of young cotyledons enhanced faster root growth in Douglas fir seedlings [96,97]. In contrast, polycotyly in angiosperms “has apparently been treated in the past as a rare and somewhat trivial abnormality” [4], and the literature from teratological studies, as mentioned above, has revealed that a variable proportion of angiosperm polycotyly was aborted, had abnormal growth, and/or did not bear seeds (e.g., [62,98]). Such a reduced seedling fitness could be explained if the cotyledon number is considered a fixed or threshold trait [99,100], as the trait might be largely canalized [101,102] and may have a threshold (or evolutionary constraint) buffered against a certain amount of genetic and environmental change [90]. However, it cannot be ruled out that angiosperm polycotyly is a useful trait for studying the evolutionary processes responsible for long-term evolutionary stasis and change [103]. Interestingly, a theoretical study argued that polyembryony can compensate for the loss in the fitness of the maternal genotype and progeny and gained some empirical support from *Citrus* plants [104]. If so, we can question what the role of polycotyly is in such compensation, as the germination of polyembryonic coniferous seeds has been associated with the occurrence of polycotyly [105].

How polycotyly evolves as a quantitative trait is also another interesting, but largely under-explored, question. The extent and distribution of polycotyly in gymnosperms and angiosperms, summarized in Table 1, seem to suggest that polycotyly is a convergent trait. It seems to occur independently across diverse phylogenetic lineages, while two cotyledons represent a conserved ancestral trait inherited from a common ancestor. Such a convergent evolution would be better explained if specific dating of polycotyly occurrences in different aged phylogenetic lineages of flowering plants was carried out. The foregoing literature also confirms that it is a heritable trait and that its expression is highly dependent on the environment. Based on the theories of evolutionary quantitative genetics [106,107], one could reason that polycotyly is an evolvable trait. Some studies have shown that polycotyly in forest trees is adaptive, as variations in cotyledon number were associated with geographical origins [88,108,109]. As mentioned above, differences in angiosperm polycotyly were found to be associated with different environments and seasons, and cotyledon counts varied among seedlings from different branch positions. Given the extent of the variation in cotyledon number and the level of estimated heritability, as described above, one could also reason that polycotyly in conifers should be more evolvable than angiosperm polycotyly. However, this reasoning calls for more research.

Theories also exist in the literature concerning the evolutionary status of polycotyly. Duchartre [7] proposed a thesis from his anatomic studies that polycotyly is derived from dicotyledony. Based on his studies of the vascular anatomy of many conifer seedlings, Hill and de Fraine [11,12,13] supported the thesis with an explanation of cotyledon splitting. From his studies on cotyledon fusion in several conifers, Buchholz [110] concluded that polycotyly is the primitive condition, and cotyledon fusion has given rise to a reduced number of cotyledons. The researcher further predicted that the cotyledon number among conifers would decrease to the limit of three or two [111]. Similarly, Woltz [112] reasoned from his cotyledon study of 55 conifer species that the assayed species, generally with multi-nerve cotyledons, may represent the evolutionary end of a phylum. These evolutionary predictions are worthy of further evaluation for a better understanding of polycotyly evolution.

### 3.5. Breeding

De Vries [19,20] was the first to describe the practical possibilities in plant breeding by selecting plants for polycotyly in his publication *Association of Characters* on plant breeding. Specifically, he wrote: “By choosing the stray seedlings with three seed leaves … the chance of producing monstrosities on a given space being proportionally increased.” He further illustrated his idea with tricotyly selection in a few wild species and acquired up to 64% polycotyly in *Antirrhinum majus* over six generations and an intermediate race of sunflower flowers, yielding from 50 to 95% polycotyly after 10 generations of selection. Similarly, Cervidalli [113] reported some races of beans (*Phaseolus vulgaris* L.) with 93.83% polycotyly over five years of cultivation; Stubbe [30] produced an F6 hybrid of *A. majus* × *A. tortuosum* containing 92–97% tricotyledonous individuals; and Reynard [16] increased the seedlings of a hybrid of *Lycopersicon pimpinelliforlium* × *L. esculentum* from 1% to 94.6% polycotyly by the fifth generation. However, no reports are found of the release of a breeding line with 100% phenotypic polycotyledony. Despite this, literature also exists on correlated responses to tricotyly selection [79,114,115] and associations between cotyledons and other traits (e.g., [22,98,116,117]). Based on the theory of correlated responses to polygenic selection, Haskell [118] promoted the use of seedling morphologies such as polycotyly as a tool to facilitate plant breeding, particularly forest tree breeding. For example, the cotyledon number was used to verify hybrids between noble fir and California red fir [119] and the hybrid parentages of Douglas fir seedlings [89].

According to Haskell [3], Litovchenko [120] promoted tricots for better use in Soviet agriculture and considered tricots to be of great economic value, as they represent a group of high productivity. For example, tricots in sugar beet occurred only among the larger seed clusters, and tricot castor oil plants were more vigorous and fertile than dicots. Such observations of this property of tricots (or its associations with other traits) have not been lacking for many other species. For example, Griffith [121] reported that tetracotylous and tricotylous seedlings of *Cupressus lusitanica* Mill. had significantly greater one-year heights than dicotyledonous seedlings. Venkatesh and Sharma [122] found tricotylous *Eucalyptus* seedlings more vigorous than dicotylous seedlings. However, there have been no comprehensive investigations of tricot characteristics of agricultural or pharmaceutical interest in a given species [22]. Thus, the breeding potential of polycotylous plants remains largely unknown.

However, there is existing literature on the exploration of polycotyly use in plant breeding [22,115,118,123,124], largely on the development of polycot genetic markers and tricot germplasms. Rajora and Zsuffa [125] explored the potential of using atypical *Populus* seedlings in poplar breeding and found that tricotylous seedlings had more branches and thicker stems than dicotyledonous seedlings and were also hardier in winter. Rick et al. [123] developed a polycot-based tomato seedling marker through extensive testcrossing, and Madishetty et al. [126] identified another tomato polycot allele through genetic mapping of a polycot mutant, but none of these two polycot markers were applied to assist with tomato tricot breeding. Taylor and Mundel [127] released multiple-cotyledon red clover genetic marker stock: L38-1485. Hu et al. [124,128] developed a sunflower germplasm line called “Tricot” with up to 50% tricotyly penetrance (USDA-GRIN PI642084). Ran et al. [22] developed two elite tricotylous lines of sand rice (*Agriophyllum squarrosum* (L.) Moq.) via the classical breeding method from four natural populations in the deserts of China. A similar characterization of 132 yellow mustard accessions was made for explorable tricot breeding, and 46 accessions were found with tricot occurrence frequencies ranging from 0.6 to 11.3% in the germinated seeds [82]. Despite these marker and germplasm developments, there is no report of the development of a specific breeding program to explore and utilize polycotyly.

A successful breeding program for a trait of interest is highly dependent on the genetic heritability (or genetic variance) of the trait, for which germplasm can be effectively selected. As demonstrated in wild radish by Conner and Agrawal [90], however, the additive genetic variance in cotyledon number present in the natural populations of an angiosperm species may be generally low, which would limit the power of selective breeding for polycotyly. Specifically, in their garden study with 1857 wild radish plants from 75 paternal half-sibling families, only 4.8% (or 89) plants were found to have cotyledon numbers less or greater than two. More research on other angiosperm species is needed to understand the generality of low genetic variance in cotyledon numbers.

## 4. Future Exploration in Polycotyly

From the foregoing review (although not exhaustive), one can conclude that we do not know much about polycotyly yet, even though it has been widely recorded in the literature for the last two centuries. The old statement on polycotyly as a rare, abnormal phenomenon in seedlings still holds for angiosperms (Supplementary Material Section A). Cotyledon numbers and features are used to classify flowering plants, but molecular phylogenetic analyses since the 1990s have largely discredited such classification of ancestral affinity. A marked advance in the research was the application of genetic mutants of cotyledons as tools for cloning and characterizing the genes regulating cotyledon development. Many genes and regulatory pathways were discovered, allowing for better elucidation and understanding of cotyledons’ origins and development. However, little is known about the character of polycotyly per se: its origin, development, genetics, and evolution. Less explored is its potential value, if there is any, for plant improvements through breeding.

Several types of research below may be beneficial for us to discover, explore, and utilize polycotyly as a quantitative trait:

A. The effort to identify polycotyly in other plant species should continue to obtain better coverage of its occurrence in seed-bearing plants.

B. Genomic analyses of polycotyly can allow us to determine its genetic basis. Genome-wide association analyses can determine how many genes are involved in their effects and where these genes are located in a chromosome. 

C. Transcriptomic analyses of polycotyly (of various natures) in many species would facilitate our search for unique patterns of gene expression and the identification of regulatory families and pathways conditioning cotyledon development. These analyses, along with physiological investigations, would expand our understanding of cotyledon origin, function, and development.

D. More informative genetic analyses of polycotyly are needed to investigate its heritability in natural populations and controlled environments. These analyses can facilitate the estimation of genetic variance and breeding for cotyledon numbers. It would also advance our knowledge of the evolvability of polycotyly.

## 5. Conclusions

Our literature search enhanced the documentation of records on polycotyly for 342 angiosperm species of 80 families and 160 gymnosperm species of 6 families. The credibility of cotyledon-based classification of flowering plants in terms of ancestral affinity has been largely questioned on the basis of molecular phylogenetic analyses. The application of genetic mutants of cotyledon as tools for cloning and characterizing the genes conditioning cotyledon mutants has improved our understanding of cotyledons’ origins and development. No breeding lines with a 100% frequency of polycotyly have been developed. Research is needed to explore the nature, development, genetics, evolution, and potential use of polycotyly as a quantitative trait.

## Figures and Tables

**Table 1:** List of 86 taxonomic families with the numbers of known species and reported species with polycotyly in eudicots of angiosperms and gymnosperms.

	Known	Species with		Known	Species with
Family	Species	Polycotyly	Family	Species	Polycotyly
***Angiosperms-Eudicots***			***Angiosperms-Eudicots***		
Acanthaceae	4000	1	Malpighiaceae	1315	1
Actinidiaceae	360	1	Malvaceae	4225	7
Adoxaceae	225	1	Martyniaceae	16	1
Aizoaceae	1900	1	Meliaceae	600	1
Amaranthaceae	2040	11	Menyanthaceae	60	1
Anacardiaceae	860	3	Moraceae	1180	2
Apiaceae	3575	8	Myrtaceae	5950	4
Apocynaceae	5100	3	Nitrariaceae	19	2
Araliaceae	1650	3	Nyctaginaceae	400	1
Asteraceae	24,700	27	Olacaceae	180	1
Balsaminaceae	1000	3	Oleaceae	790	1
Berberidaceae	700	1	Onagraceae	656	4
Betulaceae	167	2	Orobanchaceae	1960	2
Boraginaceae	2535	2	Oxalidaceae	570	1
Brassicaceae	3628	16	Papaveraceae	775	5
Cactaceae	1750	6	Phyllanthaceae	2050	1
Campanulaceae	2300	4	Pittosporaceae	245	2
Cannabaceae	100	1	Plantaginaceae	1900	8
Caprifoliaceae	825	2	Plumbaginaceae	725	6
Caricaceae	35	1	Polemoniaceae	350	2
Caryophyllaceae	2625	11	Polygonaceae	1200	2
Cleomaceae	346	1	Primulaceae	2790	7
Combretaceae	530	3	Proteaceae	1660	25
Convolvulaceae	1660	2	Ranunculaceae	2346	15
Cornaceae	85	1	Resedaceae	107	1
Crassulaceae	1400	1	Rosaceae	2950	8
Cucurbitaceae	965	4	Rubiaceae	13,620	2
Degeneriaceae	2	1	Rutaceae	2070	5
Ebenaceae	800	1	Salicaceae	1220	2
Elaeagnaceae	60	1	Sapindaceae	1860	6
Ericaceae	4250	2	Sapotaceae	1273	1
Euphorbiaceae	6252	7	Scrophulariaceae	1830	2
Fabaceae	19,500	39	Solanaceae	2600	11
Fagaceae	927	2	Theaceae	240	1
Garryaceae	25	1	Vitaceae	910	1
Geraniaceae	830	2	*Total*	171,983	342
Gesneriaceae	3540	1			
Grossulariaceae	150	1	***Gymnosperms***		
Haloragaceae	145	1	Araucariaceae	41	3
Juglandaceae	50	3	Cupressaceae	140	31
Lamiaceae	7530	7	Pinaceae	250	122
Linaceae	255	1	Podocarpaceae	156	1
Loranthaceae	1050	6	Sciadopityaceae	1	1
Lythraceae	620	2	Taxaceae	30	2
Magnoliaceae	294	2	*Total*	618	160

## Data Availability

The data presented in this study are available in the Supplementary Materials.

## Supplementary Materials

The following supporting information can be accessed at https://doi.org/10.6084/m9.figshare.25137116 (accessed on 3 April 2024). Section A: List of species with reported polycotyly. Section B: References for reported and/or cited polycotyly.
